# Podoplanin regulates the migration of mesenchymal stromal cells and their interaction with platelets

**DOI:** 10.1242/jcs.222067

**Published:** 2019-02-25

**Authors:** Lewis S. C. Ward, Lozan Sheriff, Jennifer L. Marshall, Julia E. Manning, Alexander Brill, Gerard B. Nash, Helen M. McGettrick

**Affiliations:** 1 Institute of Inflammation and Ageing, University of Birmingham, Birmingham B15 2TT, UK; 2 Institute of Cardiovascular Sciences, College of Medical and Dental Sciences, University of Birmingham, Birmingham B15 2TT, UK; 3 Centre of Membrane and Protein and Receptors (COMPARE), Institute for Biomedical Research, The Medical School, University of Birmingham, Edgbaston, Birmingham, B15 2TT, UK; 4 Department of Pathophysiology, Sechenov First Moscow State Medical University, Moscow 119048, Russia

**Keywords:** Mesenchymal stromal cell, Podoplanin, Platelet, Migration, Endothelial cell

## Abstract

Mesenchymal stromal cells (MSCs) upregulate podoplanin at sites of infection, chronic inflammation and cancer. Here, we investigated the functional consequences of podoplanin expression on the migratory potential of MSCs and their interactions with circulating platelets. Expression of podoplanin significantly enhanced the migration of MSCs compared to MSCs lacking podoplanin. Rac-1 inhibition altered the membrane localisation of podoplanin and in turn significantly reduced MSC migration. Blocking Rac-1 activity had no effect on the migration of MSCs lacking podoplanin, indicating that it was responsible for regulation of migration through podoplanin. When podoplanin-expressing MSCs were seeded on the basal surface of a porous filter, they were able to capture platelets perfused over the uncoated apical surface and induce platelet aggregation. Similar microthrombi were observed when endothelial cells (ECs) were co-cultured on the apical surface. Confocal imaging shows podoplanin-expressing MSCs extending processes into the EC layer, and these processes could interact with circulating platelets. In both models, platelet aggregation induced by podoplanin-expressing MSCs was inhibited by treatment with recombinant soluble C-type lectin-like receptor 2 (CLEC-2; encoded by the gene *Clec1b*). Thus, podoplanin may enhance the migratory capacity of tissue-resident MSCs and enable novel interactions with cells expressing CLEC-2.

## Abstract (precis)

**Summary:** Podoplanin enhances the migration of mesenchymal stromal cells in a Rac-1-dependent manner, enabling direct interactions of subendothelial stroma with circulating platelets.

## INTRODUCTION

Podoplanin (PDPN or gp38) is a small mucin-type transmembrane glycoprotein primarily expressed on mesenchymal stromal cells (MSCs), such as type-1 alveolar cells and fibroblastic reticular cells (FRCs; reviewed by Ugorski et al., 2016), but not on vascular endothelial cells (ECs). The physiological role of podoplanin in tissue-resident MSCs is not well understood, but it is believed to play a role in embryonic development of the lymphatic system and lungs, vascular integrity, platelet activation (e.g. Suzuki-Inoue, 2017), cellular migration and epithelial–mesenchymal transition (Martin-Villar et al., 2015). Some MSC types show natural variation in podoplanin expression between individual cell donors (Sheriff et al., 2018), and in some cases within specific MSC types (e.g. different fibroblast subpopulations). Moreover, expression of podoplanin can be modified by MSC responses to inflammatory mediators (Sheriff et al., 2018; Croft et al., 2016), and is often upregulated in inflamed tissues (Del Rey et al., 2014; Inoue et al., 2015; Croft et al., 2016; Hitchcock et al., 2015) and cancer (Schacht et al., 2005), where it could contribute to further pathology. For instance, in cancer, high expression correlates with increased invasion and tumour metastasis, and a poorer prognosis (reviewed by Dang et al., 2014).

Podoplanin is enriched in filopodia-like structures and coupled to the actin cytoskeleton through ezrin/radixin/moesin (ERM) family proteins allowing it to regulate cell shape and movement (Martín-Villar et al., 2006). It can enhance cellular migration of MSCs, including fibroblast-like cells (Suchanski et al., 2017) and specific ECs (lymphatic endothelial cells; LECs) (Navarro et al., 2011; Langan et al., 2018), as well as providing directional cues in epithelial cells (Martin-Villar et al., 2010). However, studies examining the involvement of Rho family of GTPases and their downstream effector proteins have yielded conflicting findings. Podoplanin signalling through ROCK (herein referring to both ROCK1 and ROCK2) has been reported to enhance the ability of cancer-associated fibroblasts (CAFs) to invade collagen matrices *in vitro* (Neri et al., 2015) and fibroblast-like cell lines to migrate across Transwell filters (Suchanski et al., 2017). VEGF-induced LEC migration (Langan et al., 2018) and FRC contraction of collagen (Astarita et al., 2015) has also been shown to be dependent on RhoA. Conversely, blocking either RhoA or ROCK promotes, rather than inhibits, the invasion of the podoplanin-overexpressing MCF-7 breast cancer cell line into collagen gels (Wicki et al., 2006; Petrie et al., 2012). Much of the evidence linking podoplanin with cellular migration has been gleaned from studies on tumour or lymphoid stromal cells. As a result, our understanding of its function in MSCs from healthy tissues is limited.

Podoplanin is the endogenous ligand for C-type lectin-like receptor 2 (CLEC-2; encoded by the gene *Clec1b*), which is expressed by platelets, dendritic cells and circulating CD11b^+^ Gr-1^+^ myeloid cells (Lowe et al., 2015). CLEC-2 ligation of podoplanin has been reported to negatively regulate MSC functions, reducing FRC contraction (Astarita et al., 2015). By contrast, we recently reported that MSCs expressing podoplanin induced CLEC-2-mediated platelet activation, causing platelet depletion when administered as a systemic cell therapy (Sheriff et al., 2018). Similar platelet activation and subsequent thrombus formation has been attributed to interactions between podoplanin-expressing perivascular cells and circulating platelets in murine models of deep vein thrombosis (Payne et al., 2017) and *Salmonella* infection (Hitchcock et al., 2015), and in patients with podoplanin-positive brain tumours (Riedl et al., 2017). Indeed, MSC–platelet interactions and their implications in malignancy have been extensively reviewed (Yan and Jurasz, 2016). More recently, a new protective role for the podoplanin–CLEC-2 axis has been described, where platelets aid recruitment of podoplanin-expressing macrophages that control bacterial-induced murine sepsis (Rayes et al., 2017). Others have shown that podoplanin–CLEC-2 interactions regulate the integrity of endothelial–endothelial and endothelial–stromal cell junctions (Herzog et al., 2013), which could explain the reduced leakage of platelets from ‘hyper-permeable’ inflamed vessels *in vivo* (Boulaftali et al., 2013). However, the cells expressing podoplanin and CLEC-2 are usually located in different anatomical compartments (tissue versus blood respectively) separated by the blood vascular ECs. Moreover, the mechanisms by which podoplanin-expressing perivascular MSCs ‘breach’ the endothelial layer to interact with circulating platelets in the absence of vessel damage remains unclear.

Comparing podoplanin-positive and podoplanin-negative umbilical cord MSCs, we studied the ability of podoplanin to regulate the motility of subendothelial MSCs and their interaction with platelets. Expression of podoplanin enhanced MSC migration in a Rac-1-dependent manner, while ROCK and RhoA–RhoC had opposing roles in regulating the podoplanin-independent component of MSC migration. From their subendothelial location, podoplanin-expressing MSCs are located in close proximity to ECs *in vivo* and appear to protrude into a monolayer of resting ECs to capture flowing platelets through interactions with CLEC-2, inducing their activation and aggregation *in vitro*. We propose that podoplanin expression imparts a pro-migratory phenotype in MSCs, facilitating their intravasation and interactions with circulating platelets, and therefore may contribute to vascular integrity and inflammation-induced thrombosis (thrombo-inflammation).

## RESULTS

### Expression of podoplanin regulates MSC migration

**Fig. 1.**
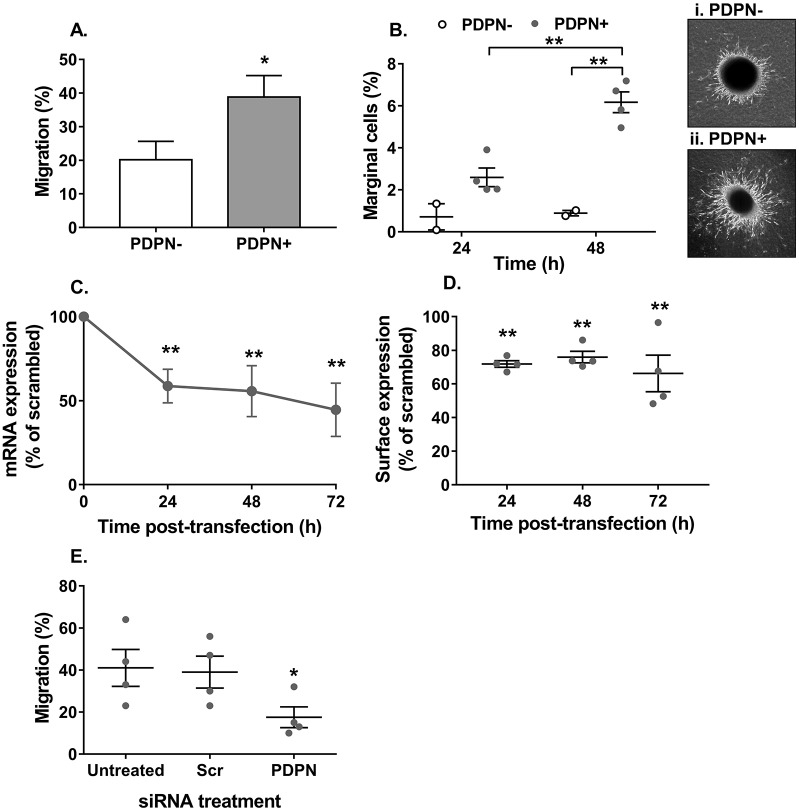
**Podoplanin expression regulates MSC migration.** Podoplanin-negative (PDPN−) and -positive (PDPN+) MSCs were seeded (A) onto an 8 µm pore filter, or (B) in a spheroid cultured on a collagen matrix. In A, migration was assessed at 48 h by counting the number of cells detached from the upper and lower chamber. Data are expressed as the number of cells in the lower chamber as a percentage of the total cell count for both chambers. *P<0.05 by unpaired *t*-test. In B, migration was assessed at 24 h and 48 h by counting the number of cells migrating along the surface of the collagen matrix and expressing it as a percentage of the total number of cells seeded. Two-way ANOVA: *P*<0.05 for time, *P*<0.01 for podoplanin status; **P<0.01 by Bonferroni post-test. Representative images of (i) PDPN− and (ii) PDPN+ MSCs migrating away from the spheroid and into the collagen gel. (C–E) Podoplanin-positive MSCs transfected with Lipofectamine (untreated) containing non-specific siRNA (Scr) or siRNA against podoplanin (PDPN) were seeded onto an 8 µm pore filter. In C, podoplanin mRNA expression was assessed over 72 h by qPCR and data are expressed as the 2^−ΔCT^ normalised to the percentage of the scrambled control. Two-way ANOVA: *P*<0.05 for time, *P*<0.001 for siRNA duplex; **P<0.01 by Dunnett post-test compared to scrambled control. In D, podoplanin protein expression was assessed over 72 h by flow cytometry and data expressed as the MFI normalised to the percentage of the scrambled control. Two-way ANOVA: *P*<0.01 for siRNA duplex; *P*>0.05 for time, **P<0.01 by Bonferroni post-test compared to scrambled control. In E, the effect of podoplanin siRNA on migration was assessed at 48 h by counting the number of cells detached from the upper and lower chamber. Data are expressed as the number of cells in the lower chamber as a percentage of the total cell count for both chambers. One-way ANOVA: *P*<0.05; *P<0.05 by Dunnett’s post-test compared to untreated control. Data are mean±s.e.m. from (A) *n*=8 PDPN− MSCs and *n*=7 PDPN+ MSCs, (B) *n*=2 PDPN− MSCs and *n*=4 PDPN+ MSCs, and (D,E) *n*=4 independent experiments using different biological donors for each cell type in each independent experiment. In C, data are mean±s.d. for *n*=3 independent experiments each using different biological donors for each cell type, where each data point per experiment was derived from the mean of three technical replicates.

We have recently reported that umbilical cord MSCs have a natural variation in their expression of podoplanin (Sheriff et al., 2018). Initially, we investigated the effect of podoplanin expression on the migratory capacity of MSCs. Podoplanin expressing MSCs displayed an enhanced ability to migrate, with significantly greater numbers transiting through a Transwell filter (Fig. 1A) and migrating along a collagen gel (Fig. 1B) when compared to MSCs lacking podoplanin. To ascertain whether this was a direct effect of podoplanin, we used siRNA to knockdown its expression in MSCs. Podoplanin siRNA caused a partial, but significant, knockdown of podoplanin mRNA and protein expression (Fig. 1C,D), which was sufficient to significantly reduce MSC migration when compared to that of podoplanin-positive MSCs treated with scrambled siRNA (Fig. 1E). Migration of podoplanin-negative MSCs was unaffected by podoplanin siRNA treatment (Fig. S1A). Furthermore, cross-linking the extracellular domain of podoplanin had no effect on MSC migration (Fig. S1B). Collectively, these data indicate that the expression of podoplanin enhances the migratory capacity of MSCs.

### Podoplanin mediates MSC migration through Rac-1

**Fig. 2.**
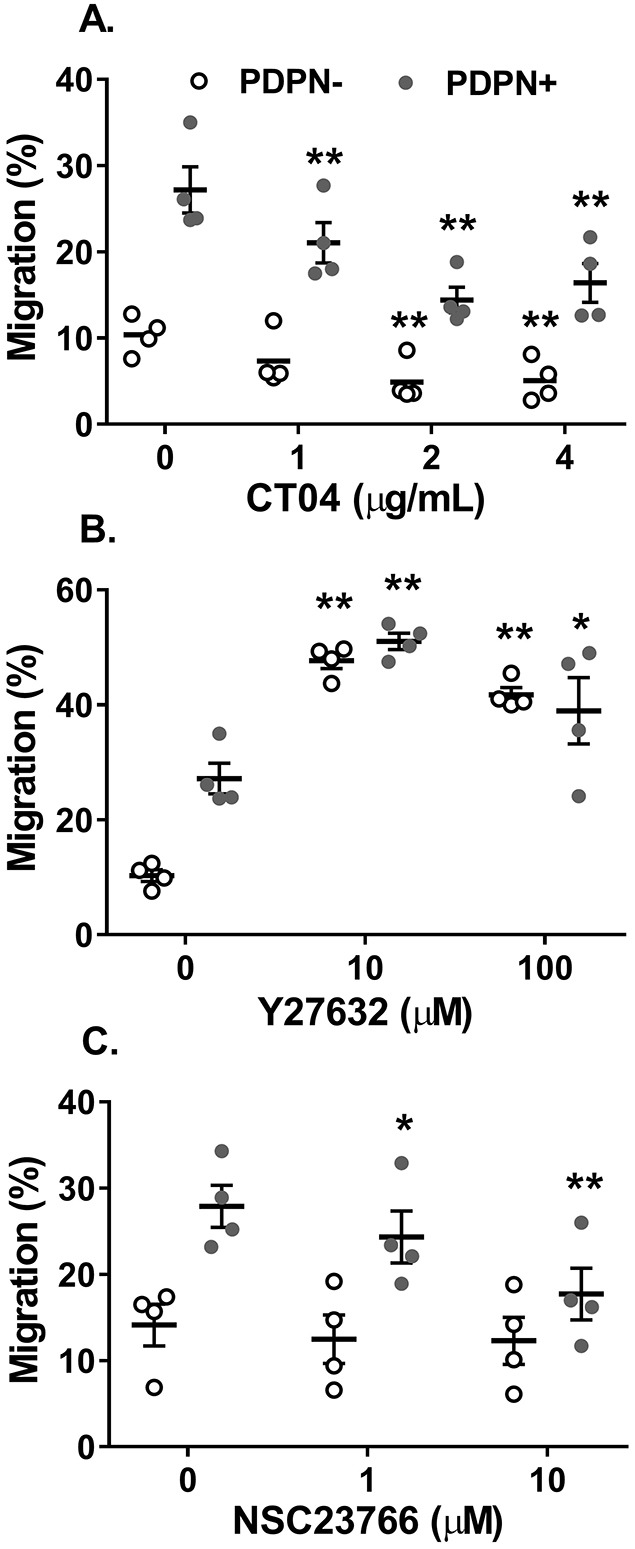
**Podoplanin-dependent MSC migration is mediated through Rac-1.** Podoplanin-negative (PDPN−) and -positive (PDPN+) MSCs seeded onto an 8 µm pore filter were treated with inhibitors against (A) RhoA–RhoC (CT04), (B) ROCK (Y27632) or (C) Rac-1 (NSC23766) over a range of concentrations. Migration was assessed at 48 h by counting the number of cells detached from the upper and lower chamber. Data are expressed as the number of cells in the lower chamber as a percentage of the total cell count for both chambers. In all cases, one-way ANOVA: *P*<0.01; *P<0.05, **P<0.01 by Dunnett’s post-test compared to untreated control for each MSC type. Data are mean±s.e.m. from *n*=4 independent experiments using different biological donors for each cell type in each independent experiment.

Next, we investigated the role of Rho family GTPases and the ROCK downstream effector protein in mediating podoplanin-induced MSC migration. MSC migration was significantly impaired when cells were treated with a non-specific inhibitor against RhoA–RhoC activation (Fig. 2A). In contrast, inhibition of ROCK signalling significantly increased MSC migration (Fig. 2B). These effects were seen for cells with or without podoplanin expression. The Rac-1 inhibitor NSC23766 caused a dose-dependent reduction in the migration of podoplanin expressing MSCs, but had no effect on the migration of cells lacking podoplanin (Fig. 2C). Collectively these data suggest that all three molecules are involved in MSC migration: RhoA–RhoC and ROCK act in opposing manners to regulate podoplanin-independent migration, whereas signalling through Rac-1 is responsible for the enhanced migratory potential of podoplanin-expressing MSCs.

**Fig. 3.**
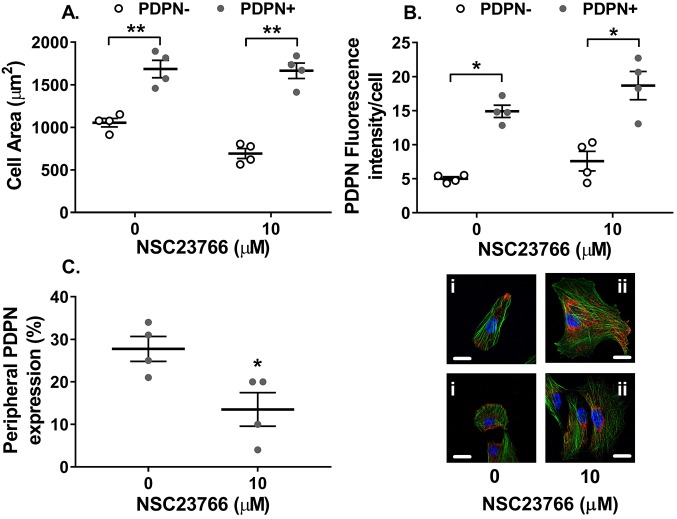
**Rac-1 inhibition alters the cellular location of podoplanin on MSCs.** Podoplanin-negative (PDPN−) and -positive (PDPN+) MSCs treated with or without the Rac-1 inhibitor NSC23766 (10 µM) for 24 h. Cellular localisation of podoplanin and F-actin was assessed by confocal microscopy. (A) Cell area was calculated as the total area of F-actin (green) staining divided by the number of nuclei (blue) and expressed as µm^2^. Two-way ANOVA: *P*<0.001 for podoplanin expression, *P*>0.05 for Rac-1 inhibition; **P<0.01 by Bonferroni post-test compared to PDPN− MSCs. (B) Fluorescence intensity of podoplanin (red) staining was assessed by using ImageJ and expressed as the integrated density per cell. Two-way ANOVA: *P*<0.01 for podoplanin expression, *P*>0.05 for Rac-1 inhibition; *P<0.05 by Bonferroni post-test compared to PDPN− MSCs. (C) The number of cells where podoplanin expression was confined to the tip of pseudopod was assessed and expressed as the percentage of total number of cells expressing podoplanin. *P<0.05 by paired *t*-test. Representative images of (i) untreated or (ii) NSC23766-treated PDPN+ MSCs, where podoplanin is red, F-actin is green and nuclei are blue. Data are mean±s.e.m. from *n*=4 independent experiments using different biological donors for each cell type in each independent experiment. Scale bars: 10 µm.

Interestingly, podoplanin-expressing MSCs seeded in microchannels were significantly larger in cell area when compared to cells lacking podoplanin (Fig. 3A). Inhibition of Rac-1 had no effect on MSC area (Fig. 3A). We also assessed podoplanin expression by immunofluorescence microscopy, and found no effect of inhibition of Rac-1 on the fluorescence intensity of podoplanin (Fig. 3B). On the other hand, it was evident that the Rac-1 influenced the cellular location of podoplanin (Fig. 3C). In podoplanin-positive MSCs, 30% of podoplanin was located in the tips of pseudopods (Fig. 3C,Ci). Treatment with the Rac-1 inhibitor significantly reduced the proportion of cells just expressing podoplanin in the tips of pseudopod to 10% (Fig. 3C), causing podoplanin to be located diffusely in the membrane and cytoplasm of the cell (Fig. 3Cii). Thus, Rac-1 contributes to the membrane localisation of podoplanin in MSCs and cell spreading.

### Podoplanin-expressing MSCs can interact with flowing platelets through porous barriers

**Fig. 4.**
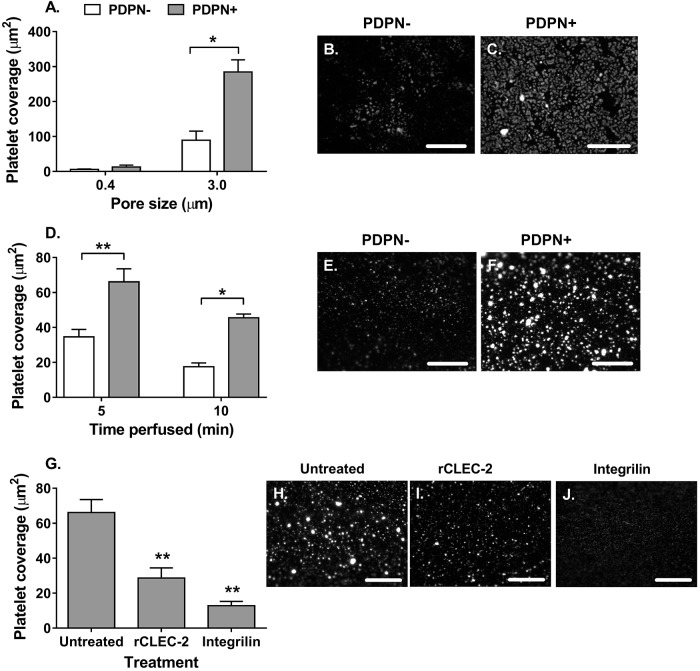
**Subendothelial MSCs interact with platelets through a filter.** Podoplanin-negative (PDPN−) and -positive (PDPN+) MSCs seeded on the basal surface of (A) 0.4 µm or (A–C) 3.0 µm pore filters for 24 h. (A) MSCs were incubated with platelet-labelled whole blood for 1 h under static conditions. Two-way ANOVA: *P*<0.01 for podoplanin status, *P*<0.001 for pore size; *P<0.05 by Bonferroni post-test compared to PDPN− donors. Representative images of (B) PDPN− MSCs and (C) PDPN+ MSCs on 3.0 µm pore filters. (D) Platelet-labelled whole blood was perfused over the apical surface of filters coated with MSCs on the basal surface for 5 or 10 min. Two-way ANOVA: *P*<0.001 for podoplanin status, *P*<0.01 for time; *P<0.05, **P<0.01 by Bonferroni post-test compared to PDPN− donors. Representative images of (E) PDPN− MSCs and (F) PDPN+ MSCs at 10 min. (G) MSCs were left untreated or treated with recombinant CLEC-2 (rCLEC-2) prior to perfusion of platelet-labelled whole blood over the over the apical surface of filters coated with MSCs on the basal surface for 5 min. Additionally, platelet-labelled whole blood was treated with the αIIbβ3-integrin small molecular inhibitor integrilin immediately prior to perfusion. One-way ANOVA: *P*<0.01; **P<0.01 by Dunnett’s post-test compared to the untreated MSC control. Representative images of platelet interactions with (H) untreated MSCs, (I) MSCs pre-treated with rCLEC-2 and (J) integrilin-treated whole blood binding to untreated MSCs. In all cases, platelet adhesion and aggregation were assessed using ImageJ particle analysis and expressed as platelet coverage in µm^2^. Data are mean±s.e.m. from (A,G) *n*=4 and (D) *n*=3 independent experiments using different biological donors for each cell type in each independent experiment; *n*≥5 fields of view were analysed per treatment group. Scale bars: 200 µm.

Increased motility and spreading of podoplanin-expressing MSCs might provide a means by which they are able to interact with circulating platelets across an endothelial barrier *in vivo*. We, therefore, examined whether podoplanin-expressing MSCs could protrude across the wall of ‘vascular’ constructs and interact with flowing platelets through CLEC-2. First, MSCs were seeded on the basal surface of 3 μm porous filters (where the pores were too small for the whole MSC cell body to cross; Fig. S2A), and labelled platelets in whole blood were added to the apical surface of the filter under static or flow conditions. Individual platelets were observed scattered across the surface of filters with podoplanin-negative MSCs (Fig. 4B,E). In contrast, greater levels of platelet adhesion were observed on the surface of filters with podoplanin-positive MSCs, and these platelets tended to clump together forming platelet microthrombi (Fig. 4C,F). Platelet binding and aggregation, indicative of activation, occurred on the apical surface of 3.0 µm pore filters in both static (Fig. 4A–C) and flow conditions (Fig. 4D–F). Under both conditions, platelet binding and aggregation was significantly greater on apical surface of filters with podoplanin-expressing MSCs underneath compared with cells lacking podoplanin (Fig. 4A,D). Thus both podoplanin-positive and podoplanin-negative MSCs can bind platelets through a filter; however, the podoplanin-positive cells induce platelet activation, leading to platelet–platelet interactions and microthrombi formation (Fig. 4C,F). Of note, neither type of MSC was able to bind platelets when cultured on 0.4 µm pore filters (Fig. 4A), suggesting this pore size was too small to allow cellular protrusions.

Pre-treatment of MSCs with recombinant CLEC-2 significantly reduced platelet coverage (Fig. 4G) and platelet microthrombi (Fig. 4I) compared to untreated controls (Fig. 4H), indicating competitive inhibition of platelet activation. To further evaluate the mechanism meditating the interaction between MSCs and platelets, whole blood was treated with a small inhibitor against αIIbβ3-integrin (integrillin), which blocks platelet–platelet interactions, but not platelet–MSC interactions (Sheriff et al., 2018). Integrillin also diminished platelet coverage (Fig. 4G), specifically reducing the number of platelet aggregates (microthrombi) observed, resulting in a uniform layer of individual platelets adhering to the filter-coated basally with MSCs (Fig. 4J). Thus, podoplanin-expressing MSCs can capture platelets from flowing blood, supporting platelet–platelet aggregation through podoplanin and activation of αIIbβ3-integrin.

**Fig. 5.**
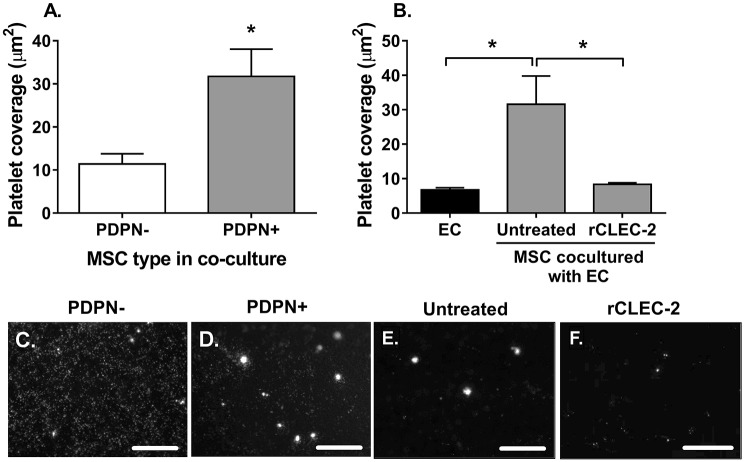
**Podoplanin-expressing MSCs can interact with platelets through endothelium.** MSC and endothelial cell co-cultures (EC:MSC) were formed on opposite sides of a porous insert. Endothelial cell (EC) mono-cultures were seeded as controls. (A) Untreated platelet-labelled whole blood was perfused over the apical surface of filters for 10 min. In all cases, platelet adhesion and aggregation were assessed by using ImageJ particle analysis and expressed as platelet coverage in µm^2^. *P<0.05 by paired *t*-test. (B) Platelet-labelled whole blood, untreated or treated with recombinant CLEC-2 (rCLEC-2), was perfused over the apical surface of filters for 10 min. One-way ANOVA: *P*<0.05; *P<0.05 by Dunnett’s post-test compared to EC:MSC co-culture. Representative fluorescent images of platelet interactions with (C) PDPN− or (D) PDPN+ MSCs in co-culture with ECs, or with PDPN+ MSC co-cultures in the (E) absence and (F) presence of rCLEC-2. Data are mean±s.e.m. from (A) *n*=3 and (B) *n*=4 independent experiments using different biological donors for each cell type in each independent experiment; *n*≥5 fields of view were analysed per treatment group. Scale bars: 200 µm.

**Fig. 6.**
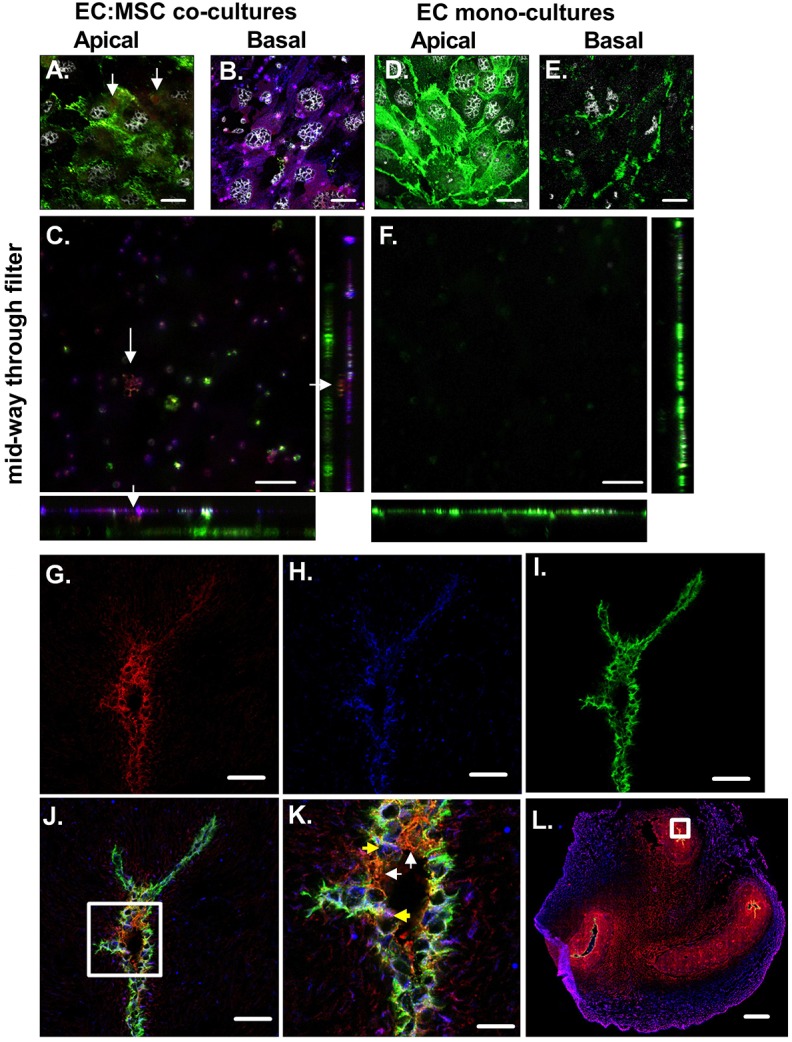
**Podoplanin-expressing MSCs can protrude through endothelium.** Cells cultured on Transwell filters (A–F) or in umbilical cord tissue sections (G–L) were stained for podoplanin, along with markers of ECs (CD31) and MSCs (CD90). (A) Monolayer of CD31-positive ECs in green on the apical surface of the filter for an EC:MSC co-culture filter. Arrows indicate red podoplanin staining on the apical surface of the filter. (B) Monolayer of MSCs expressing CD90 (in blue) and podoplanin (in red), where dual-positive cells are purple, on the basal surface of the filter for an EC:MSC co-culture filter. (C) Image obtained mid-way through the filter for an EC:MSC co-culture. Arrows indicate podoplanin from the basal MSC layer protruding up through the filter. Monolayer of CD31-positive ECs in green on the (D) apical and (E) basal surface of the filter for an EC mono-culture. (F) Image obtained mid-way through the filter for an EC mono-culture. (G) CD90 expression in red around vessels in the umbilical cord. (H) Podoplanin expression in blue around vessels in the umbilical cord. (I) CD31 expression in green around vessels in the umbilical cord. (J) CD31, podoplanin and CD90 expression around vessels in the umbilical cord. The white box represents the area magnified in panel K. (K) Zoomed image of CD31, podoplanin and CD90 expression. White arrows indicate cells positive for CD31 and CD90, but not podoplanin. Yellow arrows indicate cells appearing positive for all three markers. (L) CD31, podoplanin and CD90 expression across the entire umbilical cord section. The white box represents the zoomed in areas for G–J. Representative confocal images, obtained of using Zen Black software, are shown. Scale bars: 20 µm (A–F,K), 50 µm (G–J), 1 mm (L).

We developed a novel *in vitro* model to represent platelet interactions at the vessel wall. Here, blood vascular ECs on the apical surface of the filter were co-cultured with MSCs seeded on the basal surface Fig. S2B,C. Using this model, significantly more platelets adhered to and formed microthrombi on co-cultures incorporating podoplanin-expressing MSCs compared to those with MSCs lacking podoplanin (Fig. 5A,C,D). To determine whether platelet binding was a result of interactions with ECs or with podoplanin-expressing MSCs, we pre-treated co-cultures with recombinant CLEC-2 prior to perfusion. CLEC-2 protein significantly reduced platelet coverage (Fig. 5B) and platelet microthrombi formation (Fig. 5F) compared to untreated co-cultures (Fig. 5E), to a similar level to that seen for ECs cultured without MSCs. To account for the possibility that podoplanin might be transferred to ECs during co-culture, we assessed podoplanin expression on ECs following co-culture by flow cytometry and were unable to detect any expression by flow cytometry [podoplanin median fluorescence intensity (MFI)=0.58±0.2 mean±s.e.m., *n*=4 independent experiments with four biological donors per cell type] or confocal microscopy (Fig. 6D). Thus, a direct interaction between podoplanin-expressing MSCs and CLEC-2-expressing platelets, through the endothelial layer must be occurring.

To further assess this, we imaged the cellular organisation of ECs and MSCs on the filters via confocal microscopy. We observed a CD31 (also known as PECAM1)-positive monolayer of endothelial cells in green on the apical surface of the filter (Fig. 6A,D) and a monolayer of podoplanin and/or CD90 (also known as THY1) positive MSCs on the basal surface of the filter, where podoplanin in red colocalises with CD90 in blue (Fig. 6B). Analysis of *z*-stacks through the co-culture filter revealed regions where podoplanin extended from the basal MSC layer through the middle of the pores (Fig. 6C), indicating that MSCs are able to extend podoplanin-containing processes into the filter. We were also able to detect faint podoplanin staining on the same focal plane as CD31 on co-cultures (Fig. 6A, arrows), but not the EC monolayers (Fig. 6D), indicating that MSC-derived podoplanin was present on the apical surface on the filter upon co-culture and therefore capable of interacting with flowing platelets. Therefore, migratory podoplanin-expressing MSCs appear to be capable of protruding through the endothelium in the model vessel wall to trigger podoplanin-induced activation of captured platelets. Subsequently, we analysed expression of CD90, podoplanin and CD31 across the umbilical cord, and around the vasculature (Fig. 6G–L). We observed CD90-positive MSCs expressing podoplanin in a perivascular location, neighbouring CD31-positive blood vascular ECs (Fig. 6J,K). Expression of podoplanin by the MSCs varied, and tended to be in discrete regions of the cells rather than evenly distributed across the membrane or cell (Fig. 6H). In some cases, we were able to detect CD90 and podoplanin in contact with CD31 in the same focal plane (Fig. 6K), indicating that podoplanin and CD90 double-positive perivascular MSCs have the ability to extend podoplanin-containing processes through intact vessel walls *in vivo*.

## DISCUSSION

By using umbilical cord MSCs as a primary cell line, we have examined the relationship between the expression of podoplanin by MSCs, their migratory behaviour and ability to interact with platelets. Podoplanin expression enhanced MSC migration across a filter and over a collagen gel. Inhibition of Rac-1 altered the membrane localisation of podoplanin and, in turn, significantly reduced the migration of podoplanin-expressing MSCs, but had no effect on the migration of podoplanin-negative MSCs. Thus, Rac-1 mediated the podoplanin-induced MSC migration. We have demonstrated for the first time that podoplanin-expressing MSCs in the subendothelial space can protrude through a barrier modelling the vascular wall (including ECs) to capture flowing platelets and activate them through CLEC-2 to induce their aggregation. We propose that podoplanin expression imparts a pro-migratory phenotype in MSCs, facilitating their intravasation across the vessel wall and interaction with circulating platelets. Moreover, the upregulation of podoplanin by perivascular MSCs at sites of inflammation may contribute to the physiological regulation of vascular integrity and thrombo-inflammation. On the other hand, podoplanin presentation could have pathological consequences in the context of chronic inflammation, cancer and thrombosis.

Podoplanin has previously been reported to enhance the migration of cancer-associated MSC lines (Suchanski et al., 2017; Martin-Villar et al., 2010; Neri et al., 2015; Wicki et al., 2006; Petrie et al., 2012), and, where analysed, MSCs and ECs associated with with specialised lymphoid tissues (Navarro et al., 2011; Langan et al., 2018; Astarita et al., 2015). Although we and others agree that podoplanin confers a pro-migratory phenotype, there is no consensus on the mechanism(s) by which this is achieved. We have demonstrated that podoplanin-mediated migration is dependent on signalling through Rac-1, whereas podoplanin-independent migration is regulated via RhoA–RhoC and ROCK in umbilical cord MSCs. Indeed, our images indicate that Rac-1 is important for regulating the membrane localisation of podoplanin in MSCs. Previous groups have shown that ectopic expression of podoplanin triggers the localised recruitment of the ERM protein ezrin to the plasma membrane region involved in cell–cell contacts, promoting the internalisation or re-localisation of E-cadherin and increasing epithelial cell motility (Scholl et al., 1999; Martín-Villar et al., 2005). Rac-1 has also been reported to influence the endocytosis of E-cadherin, to control adherens junction assembly and disassembly (Jou and James Nelson, 1998; Kamei et al., 1999; Akhtar and Hotchin, 2001). Therefore, it is possible that the podoplanin–ezrin–Rac-1 complex regulates the cellular location of podoplanin in MSCs to facilitate migration. In contrast to our findings showing that Rac-1 is important for podoplanin-mediated migration, studies have indicated that RhoA or ROCK act downstream of podoplanin to influence MSC migratory patterns (Suchanski et al., 2017; Neri et al., 2015; Astarita et al., 2015; Wicki et al., 2006; Petrie et al., 2012). In lymphoid tissue-derived MSCs (FRCs) podoplanin has been shown to signal via RhoA to support cellular motility (Langan et al., 2018; Astarita et al., 2015). Although ROCK has been linked to podoplanin-mediated migration, conflicting findings have reported increased (Wicki et al., 2006; Petrie et al., 2012) and decreased (Suchanski et al., 2017; Neri et al., 2015) levels of migration in cancer-associated MSC lines treated with ROCK-blocking agents. Thus, it remains unclear whether ROCK acts to positively or negatively regulate podoplanin-mediated migration in cancer-associated MSCs. The existence of cell type-specific and/or disease-induced podoplanin signal transduction pathways may explain the differences observed in the mechanism of action of podoplanin shown in this study on healthy MSCs compared with the literature.

Additionally, the interaction of podoplanin with co-receptors or binding partners has been proposed to amplify or inhibit its signalling (reviewed by Astarita et al., 2012). For instance, CD44 reportedly interacts with podoplanin to promote cellular protrusions and provide directional cues in cancer-associated epithelial cells (Martin-Villar et al., 2010). By contrast, podoplanin interactions with CD9 in CHO cells inhibited pulmonary metastasis (i.e. cell migration) and also blocked podoplanin-mediated platelet aggregation (Nakazawa et al., 2008). In both studies, the extracellular domain of podoplanin has been suggested to be critical for cell migration. Here, we detected intracellular pools of podoplanin in both MSCs that were classified as positive and negative for podoplanin at the cell surface, yet only the podoplanin-positive MSCs display enhanced migration. Moreover, modulating surface expression or membrane location of podoplanin (i.e. the extracellular pool), either with siRNA knockdown or Rac-1 inhibition, significantly impaired the migration of podoplanin-positive MSCs. Together, these observations would suggest that it is the membrane-associated pool of podoplanin that facilitates the enhanced migration of podoplanin-positive MSCs. Further work is required to elucidate the downstream signalling mediated by podoplanin in MSCs, along with other cells, and ascertain whether it is context dependent and/or tissue specific.

It is notable that siRNA treatment could not achieve a better than ∼65% reduction in the surface expression of podoplanin on MSCs. However, this result is consistent with the published literature judging by the unquantified western blots presented (Navarro et al., 2008, 2011) and absolute flow cytometry quantification (Langan et al., 2018). Indeed, a 50% reduction in podoplanin surface expression was sufficient to significantly reduce LEC migration in response to VEGF-A back to baseline levels (Langan et al., 2018). That ∼65% of surface podoplanin is still detectable after 72 h siRNA treatment implies the presence of a long-lived portion of constitutive podoplanin. Langan et al. (2018) have postulated that there are two pools of podoplanin based on the proteins rate of turnover, a fast and slow pool, which alter the functional effects of podoplanin. In this scenario, it is possible that the long-lived pool (slow turnover pool), which is unaffected by siRNA treatment, is not linked to the migration machinery. Conversely, the fast turnover pool, which is sensitive to siRNA treatment, may be critical for the regulation of MSC migration.

Several studies have recently highlighted a protective role for podoplanin in the maintenance of vascular integrity through CLEC-2-mediated platelet activation, in the context of blood and lymphatic vessel development (Schacht et al., 2003; Bianchi et al., 2017; Fu et al., 2008), lymphocyte recirculation through high endothelial venules (Herzog et al., 2013; Boulaftali et al., 2013) and in response to infection (Hitchcock et al., 2015). Indeed, our data would support this concept. Consistent with our observations here, these studies report unevenly dispersed clumps of platelet aggregates on the vessel wall. Until this study, it had been unclear how cells in the subendothelial space would come into contact with platelets in the vessel lumen through a continuous endothelial layer. We have strong evidence that MSCs, but not ECs, present podoplanin to the luminal surface both *in vitro* and *in vivo*. Our data demonstrate that MSCs can extend podoplanin-expressing processes through pores of a filter *in vitro*. In the umbilical cord, perivascular umbilical cord MSCs are the major source of podoplanin. Interestingly, dots of CD90 and podoplanin, possibly for MSC protrusions, can be seen in contact with CD31-positive blood vascular ECs. To the best of our knowledge, the role for podoplanin on umbilical cord MSCs in the underlying physiology of the umbilical cord remains unknown. One possibility is that podoplanin and CLEC-2 interactions have a role in the maintenance of vascular integrity and vessel development, which would be crucial for the underlying biology of the umbilical cord, but further work is required in this tissue. Collectively, these data suggest MSCs can extend podoplanin-containing processes through intact EC monolayers and vessel walls, where it is able to interact with CLEC-2 on platelets in blood to induce aggregation. This process is likely to be redundant in discontinuous, sinusoidal vascular beds of the liver (Hitchcock et al., 2015) and spleen (Onder et al., 2011), where podoplanin-expressing perivascular cells (macrophages or MSCs) are exposed to the circulation allowing direct interaction with platelets. Notably, neither of these studies specifically reported the protrusion of podoplanin-expressing cells into the vessel lumens. Such interactions are likely to be highly dependent on the physical forces exerted on the individual cells, and their receptors/ligands. For instance, we have reported that LECs can capture platelets from flow over a range of shear rates; however, the optimal conditions needed, as used here, were equivalent to those of the venous network (Navarro-Núñez et al., 2015). However, the exact impact of CLEC-2 on podoplanin function in MSCs during inflammation remains to be fully determined.

In addition to the protective roles described, podoplanin is commonly upregulated in pathogenic tissues [e.g. rheumatoid joint, various cancers (Payne et al., 2017; Del Rey et al., 2014; Inoue et al., 2015; Schacht et al., 2005)] where it is believed to play a role in pathology. Indeed, patients with podoplanin-positive brain tumours had significantly reduced platelet counts and increased risk of thromboembolism (Riedl et al., 2017). Moreover, microthrombi containing podoplanin-positive tumour cells have been reported to become trapped in pulmonary vessels, enabling tumour metastasis (Kunita et al., 2007). Studies have also suggested that podoplanin–CLEC-2-induced platelet activation promotes tumour growth (Miyata et al., 2017) and facilitates epithelial-to-mesenchymal transition (Takemoto et al., 2017). In inflammation, podoplanin in the vessel wall induced thrombus formation in a murine model of deep vein thrombosis (Payne et al., 2017). The cellular source of perivascular podoplanin in that model remains to be determined, but was not thought to come from either hematopoietic or ECs (Payne et al., 2017). Hence, the role of podoplanin in eliciting either a protective or pathogenic response at the blood–stroma interface appears to be dependent on the context of its interaction with other cell types. By using umbilical cord MSCs as a primary cell line, we clearly demonstrate that the presence of podoplanin, in the absence of any disease-induced cell transformations, is sufficient to enhance cellular migration. Indeed, our evidence indicates that podoplanin is an intrinsic promoter of migration when expressed, and does not require external ligation. This is likely to be important in the perivascular niche to allow rapid MSC mobilisation to sites of angiogenesis and tissue damage, to facilitate vessel growth and tissue repair, respectively. Understanding such interactions are key to developing novel therapeutic targets based on influencing the functional properties or numbers of either CLEC-2-expressing platelets or podoplanin-expressing MSCs.

Although mesenchymal cell migration has been extensively investigated (reviewed by Eggenhofer et al., 2014), few studies have analysed the molecular machinery regulating umbilical cord mesenchymal stromal cell migration. We show for the first time that RhoA–RhoC and ROCK act in opposing manners to regulate podoplanin-independent cellular migration in umbilical cord mesenchymal stem cells, with Rho providing pro-migratory signals and ROCK inducing anti-migratory cues. By contrast, both molecules have been reported to negatively regulate bone-marrow mesenchymal stem cells; inhibiting Rho or ROCK promoted migration across transwell filters (Jaganathan et al., 2007) whereas blocking ROCK enhanced migration across ECs (Lin et al., 2013). Conversely, ROCK has also been shown to provide pro-migratory signals and positively regulate bone-marrow mesenchymal stem cell motility in response to CXCL12 (Park et al., 2017) and HIF-1α (Choi et al., 2016). Clinical trials involving systemic delivery of mesenchymal stem cells therapies are growing, with much of the therapy becoming trapped in the lungs (Schrepfer et al., 2007). Thus, there is an urgent need to improve our understanding of the migratory machinery of mesenchymal stem cell to improve their migration efficiency both in the context of MSC cell based therapies, but also as a strategy to stimulate the movement of endogenous MSCs to sites of tissue-damage and inflammation.

Podoplanin expression contributes to a pro-migratory phenotype in MSCs found in the perivascular space, lymphoid tissues (Astarita et al., 2015) and tumours (Suchanski et al., 2017; Martin-Villar et al., 2010; Neri et al., 2015; Wicki et al., 2006; Petrie et al., 2012). Perivascular MSCs signal through Rac-1, which acts to maintain podoplanin at the cell periphery and couple it to the cytoskeletal and migration machinery. Combined, these enable perivascular MSCs to protrude through the endothelial layer of the vessel wall, presenting podoplanin in the vessel lumen, which captures and activates circulating platelets in a CLEC-2-dependent manner. Physiologically this may function to maintain vascular integrity by localising MSCs to the perivascular space. However, this can also have detrimental effects in, for example, cancer by contributing to metastasis. Further work is required to dissect the physiological and pathological roles of podoplanin, with a focus on the signalling pathways linked to each scenario, to ascertain whether these differ in a context-dependent manner that could potentially yield therapeutic targets for, for example, metastasis.

## MATERIALS AND METHODS

### Isolation, culture and characterisation of MSCs

Umbilical cords were collected from anonymous donors with the assistance of the Birmingham Women's Health Care NHS Trust and Sandwell and West Birmingham Hospitals NHS Trust. Mesenchymal stromal cells (MSCs) were isolated from umbilical cords as previously described (Munir et al., 2016), cultured in low-glucose Dulbecco's modified Eagle's medium (DMEM) with stable levels of L-glutamine (Biosera, ZI du Bousquet, France) supplemented with 10% fetal bovine serum (FBS), 100 U/ml penicillin and 100 µg/ml streptomycin (all from Sigma-Aldrich) and used at passage 3. MSCs were dissociated with EDTA/trypsin (Sigma) as previously described (Munir et al., 2015), counted using a Cellometer (Nexcelom Bioscience Ltd, Manchester, UK) and suspended at the final desired concentration in culture medium.

### Flow cytometry for podoplanin

Expression of podoplanin was analysed using 1.25 µg/ml phycoerythrin (PE)-conjugated anti-podoplanin (clone NZ-1.3; batch 4284066; eBioscience, now Thermo-Fisher, Paisley, UK) as previously reported (Sheriff et al., 2018). Control samples were incubated with isotype-matched nonspecific conjugated antibodies. The level of expression was evaluated using a Cyan ADP flow cytometer and data were analysed offline using Summit 4.3 (both Beckman Coulter SA). Single positive MSCs were classified into cell donors that were either positive (MFI>10) or negative (MFI<10) for podoplanin (Sheriff et al., 2018).

### siRNA knockdown of podoplanin

Podoplanin-positive or -negative MSCs (4.2×10^3^ cells/mm^2^) were transfected with Lipofectamine 2000 (Thermo-Fisher) containing either two siRNA duplexes against podoplanin (50 nM; SASI_Hs01_00094891, SASI_Hs01_00192618) or a single scrambled siRNA duplex control (SIC001; all Sigma) for 6 h. Cells were washed with fresh medium and cultured for up to 72 h. Podoplanin expression was assessed by quantitative (q)PCR using Applied Biosystems Assay on Demand Primers (Thermo-Fisher), with technical replicates (*n*=3) averaged for each sample, as previously described (Munir et al., 2016) or by flow cytometry.

### Migration assay

MSCs (2.4×10^3^ cells/mm^2^) were seeded on the apical surface of 8 µm pore 12-well Transwell inserts (BD Falcon, SLS, Nottingham, UK) and allowed to migrate for 48 h. The culture medium was removed from both chambers. Cells were detached from the upper (apical) and lower (basal) surfaces of the filter using trypsin (2.5 mg/ml, Sigma) and counted using a Z2-series Coulter Counter (Beckman Coulter) as previously described (Jeffery et al., 2013). Migration was quantified by counting the number of cells in the lower chamber and expressing it as a percentage of the total number of cells counted in both chambers. In some experiments, MSCs were treated with siRNA for podoplanin or inhibitors against RhoA, RhoB and RhoC (CT04; 1, 2 or 4 µg/ml; Cytoskeleton), ROCK (Y27632; 10 or 100 µM; Sigma); or Rac-1 (NSC23766; 1 or 10 µM; Calbiochem) for the duration of the experiment. In cross-linking studies, MSCs were treated with anti-human podoplanin antibody (5 µg/ml; clone NZ-1.3; batch 4308851) for 30 min, followed by goat anti-rat IgG2a [30 µg/ml; PA1-84755; eBioscience now ThermoFisher, UK (Langan et al., 2018)] for 48 h.

### Collagen migration assay

Rat-tail collagen type 1 (2.15 mg/ml; First Link Ltd, West Midlands, UK) was mixed with 10× M199 (Gibco, Thermo-Fisher), and then neutralised by addition of 1 N NaOH on ice, as described (Jeffery et al., 2013; Munir et al., 2017). The gel was allowed to set for 15 min at 37°C, and then equilibrated for 24 h with culture medium. MSC spheroids were formed by suspending 2.5×10^4^ cells in 35 µl culture medium as a hanging droplet for 48 h, before settling onto the surface of the collagen gel. MSC migration over the collagen gel was assessed at 24 h and 48 h using an Olympus X71 Fluorescent Invert microscope enclosed at 37°C. Images were analysed off-line using AngioSys2.0 software (Cellworks, Buckingham, UK) to quantify the number of marginal cells (those cells disseminating from the edge of the spheroid on the surface of the collagen gel). Data were expressed as the number of marginal cells migrating away from the spheroid and along the surface of the collagen matrix as a percentage of the total number of cells seeded.

### Isolation and culture of ECs

Cryopreserved human dermal blood endothelial cells (BECs) were purchased at passage 2 and cultured in Endothelial Cell Growth Media MV containing Endothelial Cell Growth Media MV Supplement Mix as per the manufacturer's guidelines (PromoCell, Heidelberg, Germany). BECs were dissociated with EDTA/trypsin as described above and used at passage 5.

### Platelet isolation and pre-treatment

Venous blood was collected in tubes containing citrate-phosphate-dextrose solution with adenine at a 10:1 ratio (Sigma). Platelets were labelled by incubating whole blood with PE-conjugated mouse anti-human CD41a antibody (1.88 µg/ml; clone 5B12; batch 20025383; Dako, Cheshire, UK) for 10 min prior to use. In some experiments, 9 µM integrilin (Sigma), an inhibitor of α_IIb_β_3_-integrin (Thomas et al., 2011; Sheriff et al., 2018), was added to CD41a-labelled whole blood immediately prior to use.

### Assessing MSC–platelet interactions – static adhesion assays

MSCs (2.4×10^3^ cells/mm^2^) were cultured onto the basal surface (lower chamber) of 0.4 or 3 µm pore 6-well Transwell inserts for 24 h as described previously (Munir et al., 2016, 2017; McGettrick et al., 2017). The culture medium was removed from both chambers. Phosphate-buffered saline with Ca^2+^ and Mg^2+^ (PBS; Sigma) was added to the lower chamber. CD41a-labelled whole blood was added to the apical surface (upper chamber) of the filter for 1 h, before non-adherent cells were removed by washing. Adherent platelets were imaged using an Olympus X71 Fluorescent Invert microscope enclosed at 37°C. Adhesion and platelet aggregation were quantified by using the Particle Analysis of Fluorescence function in ImageJ (NIH) and expressed as platelet coverage in µm^2^.

### Assessing MSC–platelet interactions – flow-based adhesion assays

MSCs (2.4×10^3^ cells/mm^2^) were cultured onto the basal surface of 3 µm pore 6-well Transwell inserts as above. For some experiments, BECs (1.0×10^4^ cells/mm^2^) were seeded onto the apical surface of the filter and co-cultured with MSCs for 24 h, as described previously (Munir et al., 2016, 2017; McGettrick et al., 2017). Filters were analysed by phase-contrast microscopy prior to experimentation to confirm confluent monolayers of MSCs or ECs, or both (Fig. S2A–D). Membrane integrity and permeability was assessed through two independent measures: diffusion of 70 kDa FITC-labelled dextran (2 mg/ml; Sigma) across a blank or cell-coated filters (EC mono-cultures or EC–MSC co-cultures) over 1.5 h, where fluorescence was determined by spectrometry (Fig. S2E). Transendothelial electrical resistance (TER) across mono and co-culture filters was determined by using a Millicell ERS (Millipore) and Endohm-6 (World Precision Instruments, FL), with triplicate readings obtained for each filter, and data are expressed as mean TER in ohms for cell-coated filters once the electrical resistance of the culture medium was subtracted (Fig. S2F).

Subsequently, filters were incorporated into a parallel-plate flow chamber, such that MSCs were on the ablumenal surface and BECs were exposed to flow when included (Munir et al., 2016, 2017; McGettrick et al., 2017). For some experiments, MSCs were pre-treated with 30 µg/ml of human recombinant CLEC-2 protein (R&D systems) for 10 min prior to perfusion (Suzuki-Inoue et al., 2007).

CD41a-labelled whole blood (untreated or treated with integrillin) was perfused over the apical surface of the filter (i.e. the uncoated filter in MSC mono-cultures or the BEC layer in the co-culture construct) for 5 min at a wall shear rate of 150 s^−1^ (Navarro-Núñez et al., 2015). Digitised fluorescent recordings of five random fields were made along the centre line of the flow channel following 5 min of washout with PBS without Ca^2+^ and Mg^2+^ (Sigma) containing 5 U/ml heparin (Sagent Pharmaceuticals, Schaumburg, IL). Images were analysed off-line using the ImageJ Particle Analysis of Fluorescence function and data are expressed as platelet coverage in µm^2^.

### Confocal microscopy

MSCs (1.6×10^2^ cells/mm^2^) seeded in microchannels (µ-Slide VI^0.4^; Ibidi, Munich, Germany) were incubated with or without 10 µM NSC23766 for 24 h. Cells were fixed with formalin (10% neutral-buffered; Sigma) for 30 min, permeabilised with 0.5% Triton X-100 (Sigma) diluted in PBS for 10 min and blocked for 1 h with 1% bovine serum albumin and 10% goat serum (both from Vector Laboratories) or 10% normal horse serum (NHS). Cells were incubated with anti-human podoplanin antibody [5 µg/ml; clone NZ-1.3; batch 4308851 (Mizoguchi et al., 2018)], followed by goat Alexa Fluor 647-conjugated anti-rat-IgG antibody [10 µg/ml; A21247; batch 1834715; Invitrogen, Paisley, UK (Freeman et al., 2017)] or donkey Alexa Fluor 647-conjugated anti-rat-IgG antibody [1:500; 712-606-153; batch 129952; Jackson ImmunoResearch (Mizoguchi et al., 2018)] for 1 h each. Samples were counterstained with Alexa Fluor 488-conjugated phalloidin (16.5 nM) for 30 min and mounted in ProLong Gold Antifade Mountant containing DAPI (both from Thermo-Fisher) or ProLong Diamond Antifade Mountant (Life Technologies). Samples were imaged using Zen software on an LSM780 confocal microscope (both Zeiss) and analysed using ImageJ software. Cell area was determined as the total area of phalloidin (F-actin) staining divided by the number of nuclei per field of view, averaged and expressed as µm^2^. Podoplanin fluorescence intensity was determined using the Integrated Density Function in ImageJ, which analysed the fluorescence intensity in the Alexa Fluor 647 (podoplanin) channel and divided this by the number of nuclei per field of view. Data are expressed as integrated density/cell. Finally, the cellular location of podoplanin was assessed by counting the number of cells expressing podoplanin (i) located at the tips of pseudopod and (ii) diffuse within the membrane and intracellularly. These data were expressed as a percentage of the total number of cells analysed in all fields. Images in Fig. 3C were acquired using obtained using Zen Black software on a Zeiss LSM880 confocal microscope in Airyscan mode (both Zeiss) and analysed using FIJI (Schindelin et al., 2012).

To assess the cellular location of podoplanin, ECs were cultured alone or co-cultured with MSCs on filters for 24 h prior to the filter being washed in PBS, cut out of their holders and air dried. Membranes were fixed in acetone for 20 min at 4°C prior to staining with rat anti-human podoplanin [1:100; clone NZ-1.3; batch 4338663; Life Technologies (Mizoguchi et al., 2018)], mouse anti-human CD31 [1:100; IgG2a; clone HEC7; batch RA224581; Life Technologies (Link et al., 2011)] and mouse anti-human CD90 [1:200; IgG1; clone F15-42-1; batch 2794897; Merck Millipore (McKenzie and Fabre, 1981)] antibodies for 1 h, followed by staining with secondary antibodies Alexa Fluor 546-conjugated goat anti-rat-IgG [1:500; A11081; batch 1661229 (Quadrato et al., 2017)], Alexa Fluor 488-conjugated goat anti-mouse IgG2a [1:200; A21131; batch 1744724 (Cesnekova et al., 2016)] and Alexa Fluor 647-conjugated goat-anti-mouse IgG1 [1:500; A21240; batch 1608639; all from Life Technologies (Rao et al., 2017)] and Hoechst 33258. Filters were mounted between slides and coverslips using Prolong Diamond Antifade Mountant. Samples were imaged on the Zeiss 880 LSM confocal microscope using linear imaging and de-convoluted with appropriate colour controls using Zen Black software.

Alternatively 6 µm frozen umbilical cords sections were fixed in acetone for 20 min at 4°C. Sections were rehydrated prior in PBS, and blocked in 10% NHS in PBS prior to staining with rat anti-human podoplanin [1:100; clone NZ-1.3; batch 4338663; Life Technologies (Mizoguchi et al., 2018)], mouse anti-human CD31 [1:100; IgG2a; clone HEC7; batch RA224581; Life Technologies (Link et al., 2011)] and sheep anti-human CD90 [1:200; AF2067; batch CGJL0117011; R&D Systems (Mizoguchi et al., 2018)] antibodies for 1 h, followed by staining with secondary antibodies Alexa Fluor 546-conjugated donkey anti-sheep-IgG [1:500; A21098; batch 1776048; Life Technologies (Li et al., 2017)], Alexa Fluor 488-conjugated donkey-anti-mouse-IgG [1:200; 715-546-151; batch 130065 (Dias et al., 2018)] and Alexa Fluor 647-conjugated donkey anti-rat-IgG [1:500; 712-606-153; batch 129952; both from Jackson Immuno Research (Mizoguchi et al., 2018)] and Hoechst 33258. Slides were imaged on the Zeiss 880 LSM confocal microscope and the Zeiss AxioScan Z.1 and analysed using Zen Black software and FIJI.

### Ethics

The study was conducted in compliance with the Declaration of Helsinki. All human samples were obtained with written, informed consent and approval from the Human Biomaterial Resource Centre (Birmingham, UK), North East – Tyne and West South Research Ethics Committee, or University of Birmingham Local Ethical Review Committee.

### Statistical analysis

Data are expressed as mean±s.e.m. or mean±s.d. as stated, where at least three different MSC donors were incorporated in each independent experiment. In all cases, treatment groups were randomised prior to assay. Data were normally distributed, as assessed by Shapiro–Wilk normality test in GraphPad Prism. Multi-variant data were analysed using analysis of variance (ANOVA), followed by Bonferroni or Dunnett post-hoc tests. Univariate data were analysed using paired or unpaired *t*-tests as appropriate. *P*≤0.05 was considered statistically significant.

## Supplementary Material

Supplementary information
