# Supplementary material for: Podoplanin regulates the migration of mesenchymal stromal cells and their interaction with platelets
Source: J Cell Sci. 2019 Feb 25;132(5):jcs222067. doi: 10.1242/jcs.222067 (PMC6432720; doi:10.1242/jcs.222067)
Supplement: Supplementary information [file joces-132-222067-s1.pdf]

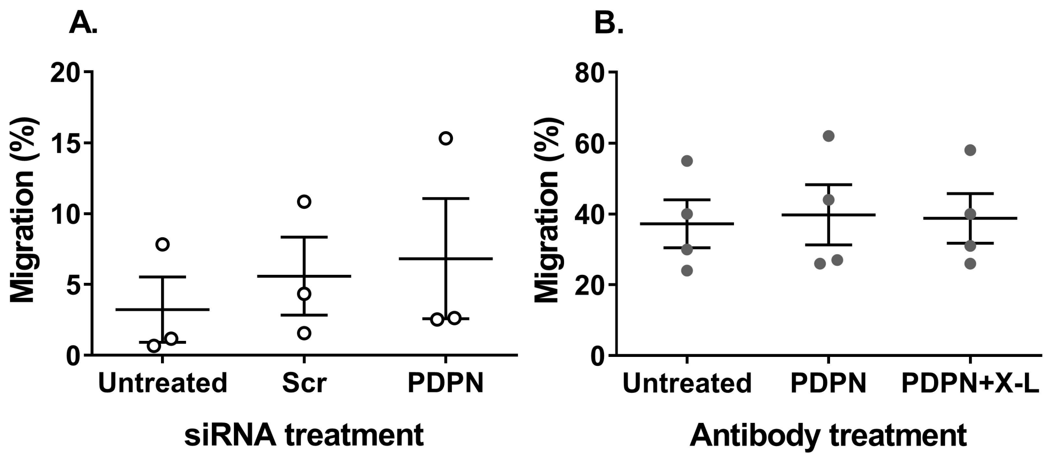

Figure S1. Effect of siRNA and podoplanin crosslinking on MSC migration (A) Podoplanin negative MSC transfected with lipofectamine (untreated) containing non-specific siRNA (scr) or siRNA against Podoplanin (PDPN) were seeded onto 8µm pore filter. **(B)** Podoplanin positive (PDPN+) MSC were seeded onto an 8µm pore filter for 1h prior to addition of anti-podoplanin antibody for 30min. MSC were subsequently treated without (PDPN) or with anti-IgG2a antibody (PDPN+X-L) for 47h to induce cross-linking of podoplanin. (A, B) Migration was assessed at 48h by counting the number of cells detached from the upper and lower chamber. Data are expressed as the number of cells in the lower chamber as a percentage of the total cell count for both chambers. One way ANOVA showed no significant effect of (A) siRNA ($p=0.23$) or (B) antibody treatment ($p=0.37$). Data are mean±s.e.m. from (A) $n=3$ and (B) $n=4$ independent experiments using different biological donors for each cell type in each independent experiment.

Journal of Cell Science • Supplementary information

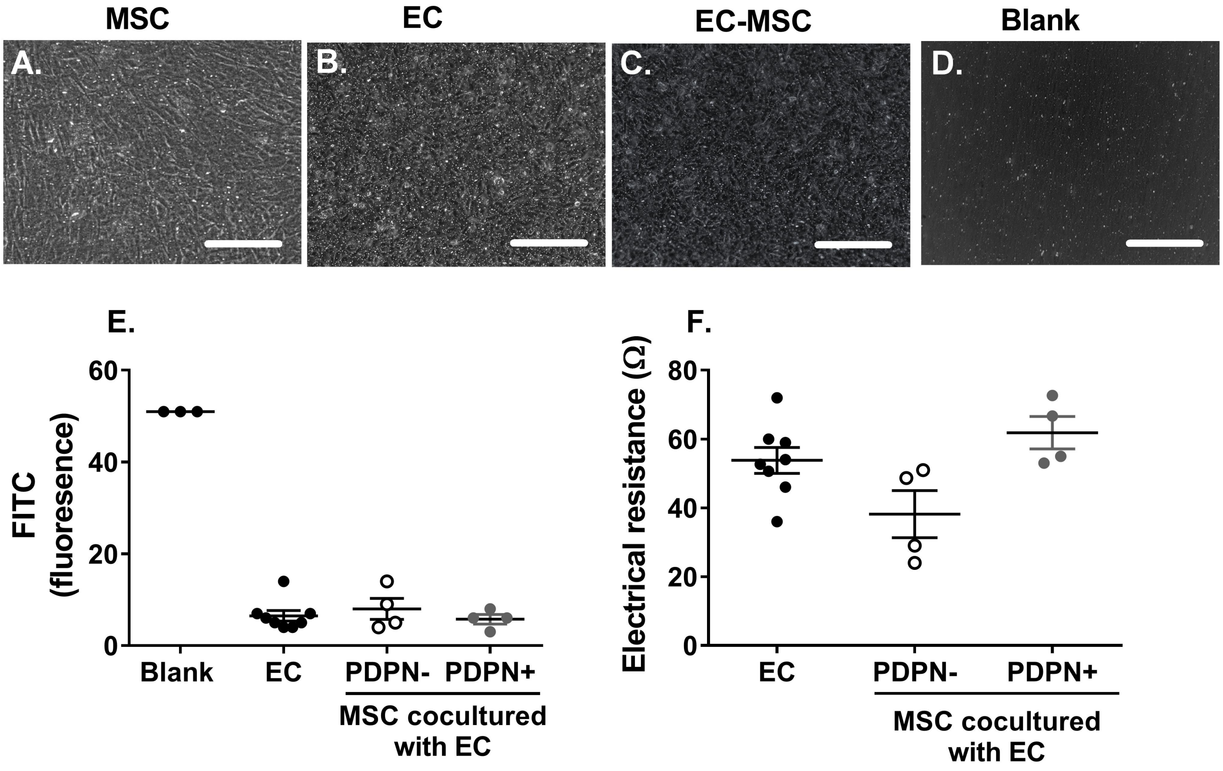

Figure S2. Validation of cell layer integrity in mono and co-cultures

MSC and endothelial cells co-cultures (EC:MSC) were formed on opposite sides of a porous insert, incorporating podoplanin positive (PDPN+) or negative MSC (PDPN-). Endothelial cell (EC) or MSC mono-cultures were seeded as controls. Representative phase contrast images of a confluent **(A)** MSC monolayer on the basal surface of the filter; EC monolayers on the apical surface of the filter when cultured **(B)** alone; or **(C)** with MSC on the basal surface of the filter. **(D)** Phase contrast image of a blank filter. **(E)** FITC-labelled dextran was allowed to diffuse through EC mono-cultures and EC-MSC co-cultures incorporating into the lower chamber over a period of 2h, after which fluorescence intensity was assessed as a measure of monolayer integrity. A blank filter was used as a control. **(F)** Electrical resistance was assessed across EC mono-cultures and EC-MSC co-cultures, normalised to the resistance of the culture media and expressed in ohms. In E and F, ANOVA showed no significant differences between EC mono-cultures and co-cultures. Data are mean±s.e.m. from n=4 independent experiments using different biological donors for each cell type in each independent experiment. Scale bar = 200µm.

Journal of Cell Science • Supplementary information
